# Editorial Notes, News and Miscellany

**Published:** 1913-07

**Authors:** 


### Editorial Notes, News and Miscellany.

A NEW SPECIALTY IN SANITARIA.—Dr. C. H. Burton of Detroit announces the opening of the West Side Sanitarium to be devoted to caring for patients suffering with locomotor ataxia.

THE NAVARRO HYPODERMIC is the quaint name of a spicy sheet issued monthly by the Navarro County Medical Society. Its motto is "Unselfishness." We have Volume 1, No. 2. It is chuck full of good things. The papers in this number are by Dr. Kelton, president; Dr. W. D. Cross, vice-president (on Eugenics); Dr. J. H. Frey and Dr. Jester. Let it came.

DR. FRANK PASCHALL, JR., now: Doctor and Mrs. Frank Paschal of San Antonio, after attending the A. M. A. meeting at Minneapolis, June 16-20, took a trip to the Thousand Islands, thence to Baltimore, where they were present at the graduating exercises at the Johns Hopkins. Their son Frank received his degree. Frank, Jr., will now remain another year at the Johns Hopkins, where he has been appointed an interne.

In the Language of the late lamented poet laureate, I am tempted to exclaim, "Bully!" on learning that Dr. A. W. Fly of Galveston has been appointed on the Board of Regents of the University of Texas. A member should be a physician, of course—why, needs not to be stated; it is obvious, and if the Governor had scraped the State with a fine-tooth comb in search of *the* one for the place he could not have made a happier selection. Dr. Fly has every qualification for the place, and his appointment will meet with the approbation of the entire profession—they all know and love him.

---

Appreciation.—Dr. R. W. Skipper, Lovelady, Texas, writes: "I send 2$ for 'Strange Case of Dr. Bruno,' your 'Dr. Jekyl and Mr. Hyde,' and another year's subscription. I have never missed a copy of The Bed-Back during all these twenty-eight years."

Dr. J. T. Benbrook, Rockwall, Texas, writes, renewing his subscription for the twenty-eighth year: "I have been a regular reader of The Red-Back since 1885 and I *couldn't think* of giving it up, having staid with you and it so long."

But here is one that comes mighty close to the fifth rib and makes me feel that, perhaps, after all, I have not "lived in vain."

Dr. Major D. Sterrett, Beckville, Texas, a subscriber *ab initio* and already paid up a year or so ahead, hands me this one: "My Dear Old Friend—My love, my congratulations go to you with this check for the twenty-ninth volume of the Journal. You have done good."

---

Practical Eugenics.—Attention is called to the advertisement in this issue of Mrs. M. E. Teats Correspondence School of Christian and Scientific Eugenics. Please drop her a postal for her prospectus, etc., naming the "Red Back."

---

The Department on Eugenics in each issue is more than worth subscription for the entire year. Kindly acknowledge receipt. Very truly yours, M. E. Daniel,
State Board of Medical Examiners.

---

Attention Is Called to nine new advertisements this issue: A. H. Robins Co., Richmond, Va.; Dawson Pharmacal Co., Dawson Springs, Ky.; the Star Ranch Sanitarium-in-the-Pines, Colorado Springs, Colo.; the Artesian Manufacturing and Bottling Co.. Waco, Texas; Austin National Bank; Jefferson Medical College; Correspondence School of Practical Eugenics; Underwood Typewriter Co., and New Orleans Polyclinic.

THE STATE BOARD OF MEDICAL EXAMINERS held its semi-annual session in Austin, June 30, ult., and examined one hundred and forty applicants. I do not know with what results.

---

TEXAS STATE BOARD OF MEDICAL EXAMINERS.—Questions for Examination in Surgery, Austin, Texas, June 24, 25 and 26, 1913:

No. 1. What are the symptoms of air embolism? Give treatment. In what region are surgical operations most liable to be followed by this condition?

No. 2. Give the symptoms of simple, compound, and compound comminuted, fracture, and treatment of each condition of tibia and fibula.

No. 3. Give symptoms of patient indicating tracheotomy; then intubation, and describe fully each operation.

No. 4. Diagnose an infective exudate of the pericardial sac, beyond controversy, and state how you are going to relieve this condition.

No. 5. Differentiate tuberculous and syphilitic synovitis.

No. 6. Give structural changes that induce symptoms of, and describe in detail, Bassini's operation for strangulated, obligue inguinal hernia.

No. 7. Differentiate concussion, compression, and congestion, of the brain.

No. 8. Give differential diagnosis of pelvic peritonitis and impaction of feces.

No. 9. Give signs and symptoms of cervical rib, and state how cervical rib produces signs and symptoms.

No. 10. How should a dissecting wound and attendant blood poisoning be treated? What is rational treatment of suppurative abscess from its initial symptom? Give symptoms and treatment of psoas abscess, and state with what condition it may be confused. —Examiner, E. B. Osborn, M. D., Cleburne. Texas.

